# Clinical Outcomes for Total Hip Arthroplasty with and without Previous Curved Periacetabular Osteotomy

**DOI:** 10.3390/jcm12020694

**Published:** 2023-01-15

**Authors:** Koichi Kinoshita, Hajime Seo, Taiki Matsunaga, Kenichiro Doi, Takuaki Yamamoto

**Affiliations:** Department of Orthopaedic Surgery, Fukuoka University Faculty of Medicine, Fukuoka 814-0180, Japan

**Keywords:** clinical outcome, curved periacetabular osteotomy, global offset, total hip arthroplasty

## Abstract

There are currently no reports on the clinical outcomes after total hip arthroplasty (THA) with previous curved periacetabular osteotomy (CPO), although the outcomes after THA with non-CPO types of periacetabular osteotomy have been reported. This study aimed to clarify the differences in clinical outcomes and radiographic features after THA with or without previous CPO. We performed a retrospective case–control with individual matching study. The participants were 10 patients with 11 hips that underwent cementless THA between October 1998 and October 2018 with previous CPO (osteotomy group). For the control group, we matched age, sex, and follow-up period, and included 32 patients with 33 hips that underwent cementless THA without previous CPO at a 1:3 ratio. The Harris Hip Score (HHS), cup size, position, and alignment, global offset (GO), operative time, perioperative blood loss, frequency of osteophyte removal, and major complications were compared between the two groups. The osteotomy group had no cases with revision surgery and dislocation. No significant differences were found between the two groups as follows: mean HHS, 94.9 points in the osteotomy group versus 92.7 points in the control group at the final follow-up; mean GO, 70.1 mm in the osteotomy group versus 71.4 mm in the control group; cup size, position, and alignment after THA; operative time; and perioperative blood loss. The frequency of osteophyte removal was higher in the osteotomy group. The take-home messages were that the clinical outcomes, including HHS, and radiographic features, including GO, after THA were equivalent in the two groups.

## 1. Introduction

Acetabular dysplasia of the hip is a known cause of hip osteoarthritis [1]. Several types of periacetabular osteotomy (PAO), such as transposition osteotomy of the acetabulum (TOA) [2], rotational acetabular osteotomy (RAO) [3], Bernese periacetabular osteotomy [4], curved periacetabular osteotomy (CPO) [5], and eccentric rotational periacetabular osteotomy (ERAO) [6], have been performed to treat symptomatic acetabular dysplasia of the hip. The reported success rates of these procedures were 94.1% to 94.2% for TOA [2,7], 78% to 96% for RAO [8,9,10], 60.5% to 82% for Bernese periacetabular osteotomy [11,12,13], 94.3% to 96.8% for CPO [5,14,15,16], and 87.3% for ERAO [17].

Unfortunately, some patients who undergo PAO subsequently require total hip arthroplasty (THA) because osteoarthritis of the hip has progressed. Restoring the condition of the inflamed hip joint through THA is a highly effective surgical intervention [18]. While the different PAO techniques all free the acetabular fragment while preserving the continuity of the posterior column, there are differences in approach, such as polygonal or curved osteotomy, and in methods to perform the osteotomy of the pelvic medial wall and pubis. These differences may impact the development of secondary osteoarthritis after PAO. Therefore, clinical outcomes after THA following previous pelvic osteotomy should be assessed separately for each PAO technique. The clinical outcomes of THA after failed TOA, RAO, and Bernese periacetabular osteotomy were reported to be equivalent to those of THA without these previous PAOs [19,20,21,22]. Meanwhile, the clinical outcomes of THA after failed ERAO were worse than those of THA without previous ERAO [23]. The novelty of these previous studies was the comparison of clinical outcomes and radiographic findings (mainly related to the pelvic side) between patients who underwent THA with different types of previous PAO and matched controls who underwent THA without previous PAO. However, these studies had several limitations, including the reliability of the records, loss to follow-up, and selection bias associated with retrospective studies, the small number of patients who underwent THA with previous PAO, the short follow-up period after THA, and, in some studies, the clinician-reported outcomes. Furthermore, no reports are available on the clinical outcomes of THA with previous CPO.

Previous PAO affects the hip joint center after subsequent THA [19,20,21,23]. Acetabular offset (AO) [24] after THA with previous PAO was reported to be significantly increased compared with that after THA without previous PAO [19,20,21,23]. However, there are no reports on global offset (GO), measured as the sum of AO and femoral offset (FO) [24], after THA with previous PAO. GO after THA is important to obtain good HHS, and therefore, it should also be investigated [25].

The objectives of the present study were to clarify clinical outcomes and radio-graphic features after THA with previous CPO. Therefore, patients who underwent THA with previous CPO were compared with matched controls who underwent THA without previous CPO. Clinical outcomes included HHS and radiographic features included GO.

## 2. Materials and Methods

### 2.1. Study Design and Patients

**Figure 1:**
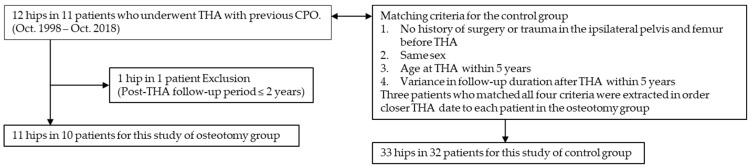
Patient selection workflow. Abbreviations: THA, total hip arthroplasty; CPO, curved periacetabular osteotomy.

We performed a retrospective review of 12 hips in 11 consecutive patients who underwent THA by three experienced hip surgeons, between October 1998 and October 2018, and had previous CPO performed by one experienced hip surgeon (Figure 1). After applying exclusion criteria of postoperative follow-up period <2 years, 1 hip in a patient with a 1-year follow-up period was excluded. Thus, 11 hips in 10 patients were included in the osteotomy group. The matching criteria for the control group were: (1) no history of surgery or trauma in the ipsilateral pelvis and femur before THA; (2) same sex; (3) age at THA within 5 years; and (4) variance in follow-up duration after THA within 5 years. Three patients who matched all four criteria were extracted to maintain a close THA date to each patient in the osteotomy group. As a result, 33 hips in 32 patients that underwent THA by three experienced hip surgeons were included in the control group. For the patient demographic characteristics, sex, age at THA, height, body weight, body mass index (BMI), pre-THA diagnosis, and follow-up duration after THA were investigated.

### 2.2. Surgical Technique

The indications for CPO included pain and limitation in daily activities for >5 months, age <65 years, lateral center-edge angle [26] <25°, and hip congruency in the abduction position on anteroposterior (AP) radiographs. In the present study, the Smith-Petersen approach [27] was used for 2 patients in 1996, the Bikini incision [28] was used for 2 patients in 1997 and 2002, and a modified Smith-Petersen approach [29], involving a shorter skin incision than that for the standard Smith-Petersen approach, was used for 7 patients between 2003 to 2007. The CPO procedure was previously reported in detail [5].

For the present study, THA was performed using four types of approaches [30,31,32,33] (Table 1). All patients in the two groups underwent THA using cementless components on both the acetabular and femoral sides. All cups had multiple screw holes. Kinectiv has a modifiable neck. S-ROM is modular with a separate proximal sleeve and femoral stem. Other femoral components were the mono-block type. On the acetabular side, the target angle for radiographic cup inclination was 40° or 45° and that for radiographic cup anteversion was 15°, 20°, or 25° in both groups.

### 2.3. Surgical Data and Clinical Evaluations

Surgical data, including operative time, perioperative blood loss calculated using the formula developed by Gloss [34], frequency of osteophyte removal, type of approach, and size of cups used, were obtained from clinical records. The Harris Hip Score (HHS) [35] was used to evaluate hip joint function prior to THA and at the final follow-up. Patients who underwent revision surgery for aseptic loosening were identified. Postoperative complications such as dislocation, infection, venous thromboembolism (VTE), and nerve palsy were determined from the medical records.

### 2.4. Radiographic Evaluations

**Figure 2:**
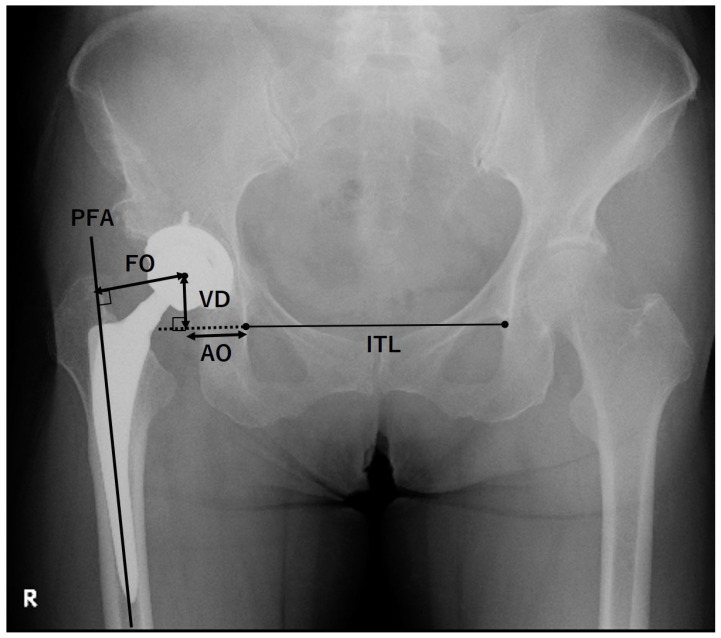
Radiographic indices measured on the anteroposterior radiographs. The interteardrop line (ITL) connects the inferior edges of both teardrops. The acetabular offset (AO) was measured as the horizontal distance from the inferior edge of the teardrop to the point where an extension of the ITL intersects with a perpendicular line drawn from the hip joint center. The vertical distance (VD) was measured as the distance from the hip joint center to the point where the extension of the ITL intersects with the perpendicular line from the hip joint center. The femoral offset (FO) was measured as the distance from the hip joint center to the intersection with the proximal femoral shaft axis (PFA). The global offset was measured as the sum of FO and AO.

Radiographic evaluations were performed on AP and cross-table lateral (CL) radiographs obtained pre-THA and at the final follow-up. The AP radiographs were taken in the supine position with a standardized tube-to-film distance (120 cm) and perpendicular orientation of the tube to the table. The CL radiographs were obtained with the contralateral hip flexed at 90°. The X-ray beam was parallel to the examination table and 45° cranial to the long axis of the trunk. The film was set perpendicular to the examination table using a film holder. The cup inclination and anteversion were measured on the AP and CL radiographs at 1 week after THA. The hip joint center, AO, vertical distance (VD), and FO were measured on the AP radiographs at 1 week after THA (Figure 2). The AO was measured as the horizontal distance from the inferior edge of the teardrop to the point where an extension of the interteardrop line (ITL), connecting the inferior edges of both teardrops, intersects with a perpendicular line from the hip joint center [36]. The VD was measured as the distance from the hip joint center to the point where the extension of the ITL intersects with the perpendicular line from the hip joint center [36]. The FO was measured as the distance from the hip joint center to the intersection with the proximal femoral shaft axis [24]. GO was measured as the sum of FO and AO. The Crowe classification [37] was evaluated on the AP radiographs before THA. Cup stability was evaluated using McPherson’s criteria [38]: grade IA, no radiolucency; grade IB, one zone of radiolucency; grade IC, two zones of radiolucency; grade II, complete radiolucent line of <2 mm in all zones; grade III, progressive radiolucent line at zone III, complete radiolucent line of ≥2 mm in all zones, or cup migration. Stem stability was evaluated using Engh’s criteria [39]: fixation by bone ingrowth, no subsidence and minimal or no radiopaque lines around the stem; stable fibrous fixation, no progressive migration and extensive radiopaque lines around the stem of ≤1 mm; unstable, progressive subsidence or migration; and at least partially surrounded by divergent radiopaque lines.

Measurements on radiographs were performed independently by two orthopedic surgeons. The observer reviewed all radiographs and performed manual measurement of each parameter three times on three on different days on a picture archiving and communication system workstation monitor (Rapideye Core; Canon Medical Systems), and the average values were calculated. The intraobserver reliabilities for the two observers were assessed for inclination (0.99 and 0.97), anteversion (0.99 and 0.99), VD (0.97 and 0.90), AO (0.97 and 0.95), and FO (0.96 and 0.97). The interobserver reliabilities were assessed for inclination (0.93), anteversion (0.94), VD (0.92), AO (0.97), and FO (0.89).

### 2.5. Statistical Analysis

The Mann–Whitney U test was used to compare patient demographic characteristics, clinical outcomes such as HHS, operative time, and perioperative blood loss, and radiographic parameters between the two groups. The Wilcoxon signed-rank test was used to compare changes in HHS and radiographic parameters within each group. The chi-square test was used to compare categorical data such as sex, complications, and Crowe classification. BellCurve for Excel version 3.22 (Social Survey Research Information Co. Ltd., Tokyo, Japan) was used for all statistical analyses. Values of *p* < 0.05 were considered statistically significant.

## 3. Results

### 3.1. Patient Demographic Characteristics

Comparisons of the patient demographic characteristics revealed no significant differences in sex, mean age at THA, BMI, Crowe classification pre-THA, diagnosis pre-THA, and follow-up duration after THA between the two groups (Table 2). There were no significant differences between the two groups for Crowe classification and pre-THA diagnosis, although only patients in the control group had a Crowe classification of II or pre-THA diagnosis of osteonecrosis of the femoral head. The mean duration of follow-up after THA was between 8.1 and 8.6 years (mid-term).

### 3.2. Surgical Data and Clinical Evaluations

No patients in the osteotomy group underwent revision surgery, while 1 patient in the control group underwent revision surgery for aseptic loosening of the stem at 1.2 years after THA (Table 3). Although the mean HHS of 47.5 points in the osteotomy group was significantly lower than the mean HHS of 60.9 points in the control group before THA, the mean HHS of 94.9 points in the osteotomy group did not differ significantly from the mean THA of 92.7 points in the control group at the final follow-up. There were no patients with dislocation, infection, or VTE after THA in either group. One patient who underwent THA using a direct anterior approach in the control group had femoral nerve palsy, but they recovered with normal knee extension during manual muscle testing at the final follow-up. Osteophyte removal at the acetabulum during THA was undertaken in 5 hips (45.5%) in the osteotomy group and 5 hips (15.2%) in the control group, with a significant difference between the groups. Screws were used in all patients in both groups. In the osteotomy group, the mean number of screws was 2.1 screws, with 1 screw in 2 hips, 2 screws in 7 hips, 3 screws in 1 hip, and 4 screws in 1 hip. In contrast, in the control group, the mean number of screws was 1.7, with 1 screw in 12 hips, 2 screws in 19 hips, 3 screws in 1 hip, and 4 screws in 1 hip. There were no significant differences in operative time, perioperative blood loss, type of approach, number of screws to anchor the cup, or cup size between the two groups.

### 3.3. Radiographic Evaluations

The mean cup inclination and anteversion, VD, AO, FO, and GO did not differ significantly between the two groups (Table 4). There were no cases with cup loosening in either group. There was 1 case in the control group with unstable stem (3.0%), but this was not significantly different to the osteotomy group.

## 4. Discussion

The present study is the first retrospective case–control with individual matching study to report the clinical outcomes and radiological findings after THA with previous CPO. Five major findings were clarified. First, there were no cases of revision THA in the osteotomy group. Second, the mean HHS at the final follow-up was equivalent between the two groups. Third, the cup position, alignment, and size were comparable between the two groups. Fourth, no cases of complications including dislocation, infection, VTE, and nerve palsy after THA were observed in the osteotomy group. Fifth, the operative time and perioperative blood loss were similar between the two groups.

In the present study, the patients in the osteotomy group did not require revision THA and did not have unstable implants. These findings are consistent with reports that patients who underwent THA after TOA, RAO, or ERAO did not require revision THA [19,20,21,23]. In contrast, Amanatullah et al. [22] reported that 2 patients who underwent THA after Bernese periacetabular osteotomy required revision THA as a result of dislocation. They further reported that there was no significant difference between the two groups regarding revision surgery, because one patient in the control group had revision THA for aseptic loosening of the acetabular component, and another patient in the control group had revision THA for problems related to a metal-on-metal articulation. We considered that previous PAO did not affect implant survival after subsequent THA.

The mean HHS at the final follow-up after THA was equivalent between the two groups in the present study. Fukui et al. [21] reported that the mean HHS of 93.2 points at a mean follow-up of 8.2 years after THA with previous RAO did not differ significantly from the mean HHS of 94.3 points at a mean follow-up of 8.7 years after THA without previous RAO. Amanatullah et al. [22] reported that the mean HHS of 95 points at a mean follow-up of 10 years after THA with previous Bernese periacetabular osteotomy did not differ significantly from the mean HHS of 93 points at a mean follow-up of 6 years after THA without previous Bernese periacetabular osteotomy. The mean HHS after THA with previous CPO compared favorably with the mean HHS after THA with previous RAO and Bernese periacetabular osteotomy.

The present study is also the first to show that previous CPO did not affect GO and mean HHS after subsequent THA. Biggi et al. [18] reported that restoring proper GO after THA was important to obtain good HHS. The present findings support this conclusion. In the present study, AO and VD from the ITL to the hip joint center also did not differ significantly between the two groups. Meanwhile, the hip joint center was reported to be located significantly laterally and superiorly after THA with previous PAO including TOA, RAO, and ERAO compared with the position after THA without these previous PAOs [19,20,21,23]. Although the mean AO in our osteotomy group was equivalent to the range of 31.2 to 38.1 mm in the previous osteotomy groups in the cited reports, the mean AO in our control group tended to be larger than the range of 28.1 mm to 31.4 mm in the control groups in the cited reports. We found that the large AO in the control group was the reason for the lack of significant difference between the two groups in the present study. The mean VD of the hip joint center was reported to be equivalent between patients with and without previous osteotomy by Fukui et al. [21] and Osawa et al. [23]. The present results are compatible with these findings. Komiyama et al. [19] recommended cup placement at the anatomic hip center, as the high decreased range of motion in THA leading to the dislocation. Furthermore, Tanaka et al. [40] reported that superior placement of the hip center caused delayed recovery of abductor muscle strength after THA. In the present study, the cup placement in the osteotomy group at an equivalent position to that in the control group was one of the factors that prevented dislocation after THA. The mean cup inclinations after THA with previous PAO (range: 38.3° to 45.8°) were reported to be equivalent to those after THA without previous PAO (range: 39.4° to 45.9°) [19,20,21,23]. The present results are consistent with these findings. However, the mean cup anteversions of 13.6° and 16.9° after THA with previous RAO and ERAO were significantly smaller than those of 16.1° and 26.1° after THA without previous RAO and ERAO [21,23]. The present results are not consistent with these findings. Amanatullah et al. [22] reported that acetabular retroversion associated with retroversion and deficiency of the posteroinferior part after PAO was related to smaller cup anteversion. These findings led us to speculate that the cup anteversion was equivalent in the two groups in our study because the transferred acetabulum after CPO did not tend to be retroverted. In our study, the cup size was comparable between the two groups. However, the mean cup sizes of 50 mm and 52.1 mm after THA with previous PAO were significantly larger those of 48 mm and 49.5 mm after THA without previous TOA and RAO [19,21]. Komiyama et al. [19] reported that a large cup size tends to be due to morphologic deformity of the acetabulum after PAO. Morphologic deformity requiring a larger size cup may be less common after CPO.

In the present study, the patients in the osteotomy group had no major complications such as infection, dislocation, VTE, and nerve palsy. Comparable results were reported after THA with previous TOA and RAO [19,20,21]. Meanwhile, Amanatullah et al. [21] reported that two patients who underwent THA after Bernese periacetabular osteotomy sustained dislocations attributable to extraarticular bony impingement and polyethylene liner wear. Ito et al. [20] reported that osteophyte removal at the acetabulum during THA was significantly more frequent in the previous RAO group compared with the control group, and that no patients had dislocation after THA. The present results are consistent with these findings. We believe that proper osteophyte resection is important to prevent dislocation after THA with prior PAO. 

Operative time and blood loss showed no significant differences between the previous PAO group and the control group according to Fukui et al. [21], Amanatullah et al. [22], and Osawa et al. [23]. The present results are consistent with these findings. Meanwhile, Komiyama et al. [19] reported that THA after previous TOA involved the removal of broad osteophytes, resulting in longer operative time and larger blood loss. Ito et al. [20] also reported that operative time was longer and osteotomy removal was more frequent in the previous RAO group compared with the control group. We speculate that the range of osteophytes requiring resection may have been narrow, which led to the equivalent findings for operative time and perioperative blood loss between the two groups in the present study. Because all patients in the two groups underwent THA with cementless implants, the type of approach and preoperative Crowe classification did not differ significantly between the two groups. Previous studies have reported the mean interval between PAO and THA to range from 3.3 to 7.5 years [41,42,43,44], which is consistent with our results. Moreover, a previous study reported fixation of cementless cups with two or three screws [21]. The present study used a comparable number of screws in the osteotomy group.

Our study has several limitations. First, the number of patients with THA after previous CPO was small. We surmise that the evaluation of larger patient populations may lead to significant differences in several parameters. Second, the clinical outcomes were evaluated over a medium-term follow-up of 8 years. In future studies, the outcomes should be evaluated for long-term follow-up of 10 years after THA. Third, the study used AP and CL radiographs. Use of computed tomography images would allow three-dimensional evaluation of GO, and more accurate evaluation of cup alignment and position, and range of osteophytes at the acetabulum. Fourth, three THA approaches were used in the osteotomy group and four THA approaches were used in the control group, although there were no significant differences between the two groups. Fifth, many types of implants were used in the two groups. However, cementless implants were used for all cases, and there were no significant differences in implant survival or GO between the groups. Sixth, Crowe classification II and ONFH were only present in the control group, although there were no significant differences between the two groups in Crowe classification or pre-THA diagnosis. This may have influenced pre-THA HHS. Finally, only the HHS, a clinician-reported outcome, was utilized in the present study. In future studies, patient-reported outcomes should also be utilized.

## 5. Conclusions

Clinical outcomes and radiographic features of patients who underwent THA with previous CPO were compared with matched controls who underwent THA without previous CPO. The clinical outcomes, including implant survival, mean HHS, and major complications, and radiological evaluations, including GO, hip joint center position, and cup inclination and anteversion, were comparable between the two groups. Future multicenter retrospective studies with a larger number of patients who underwent THA with previous CPO may help to clarify any differences in outcomes between THA with and without CPO.

## Figures and Tables

**Table 1:** Surgical data.

	Osteotomy Group	Control Group	
	(*n* = 11)	(*n* = 33)	*p*
Operative time (min)	113.5 ± 39.0 (50–180)	101.2 ± 31.8 (56–175)	0.32
Perioperative blood loss (mL)	912.1 ± 407.2 (225–1503)	828.5 ± 376.9 (27–175)	0.46
Resection of osteophyte	5 (45.5)	5 (15.2)	0.038 *
Approaches			0.54
Posterolateral	7 (63.6)	16 (48.5)	
Direct lateral	0	4 (12.1)	
Direct anterior	2 (18.2)	7 (21.2)	
Anterolateral supine	2 (18.2)	6 (18.2)	
Cup size	49.6 ± 1.5 (48–52)	49.4 ± 2.0 (46–54)	0.75
Acetabular components			
Natural hip system ^††^	1	0	
RingLock Acetabular System ^††^	1	4	
Trilogy ^††^	6	8	
Mallory Head ^††^	0	2	
G7 ^††^	0	1	
Plasmacup ^†^	1	1	
Triad HA ^‡^	0	2	
Tritanium ^‡^	0	2	
PINNACLE ^§^	0	1	
SQRUM TT shell ^||^	2	6	
Escalade ^¶^	0	1	
Nakashima ^#^	0	5	
Femoral components			
Multilock ^††^	0	1	
Natural hip system ^††^	1	0	
VerSys HA ^††^	2	3	
Kinectiv ^††^	2	1	
TaperLock Microplasty ^††^	1	5	
Bi-Metric ^††^	0	2	
Mayo conservative ^††^	0	1	
Excia ^†^	1	1	
Centpillar TMZF ^‡^	0	2	
Accolade II ^‡^	0	2	
S-ROM ^§^	0	1	
Initia ^||^	2	3	
910 PerFix HA ^||^	0	3	
OVATION Tribute ^¶^	0	1	
FS ^#^	2	7	

Data are presented as mean ± standard deviation (range), number (%), or number. Manufacturers: ^††^ Zimmer Biomet, Warsaw, IN, USA; ^†^ B. Braun, Kronberg, Germany; ^‡^ Stryker, Kalamazoo, MI, USA; ^§^ DePuy Synthes, Raynham, MA, USA; ^||^ Kyocera, Kyoto, Japan; ^¶^ Japan MDM, Tokyo, Japan; ^#^ Nakashimamedical, Okayama, Japan. * Significant difference: *p* < 0.05.

**Table 2:** Patient demographic characteristics.

	Osteotomy Group	Control Group	*p*
Number of hips	11	33	
Number of patients	10	32	
Male-to-female ratio	0:10	0:32	1.00
Age at THA (years)	51.9 ± 7.2 (34–59)	52.8 ± 6.8 (36–63)	0.73
BMI (kg/m^2^)	24.0 ± 6.0 (17.1–34.8)	24.2 ± 3.9 (18.1–34.4)	0.46
Crowe classification			0.23
I	11	29	
II	0	4	
Pre-THA diagnosis			0.17
OA	11	28	
ONFH	0	11	
Interval between CPO and THA	8.8 ± 4.4 (1.4–14.0)	N/A	
Duration of follow-up after THA (years)	8.6 ± 5.4 (3.0–17.6)	8.1 ± 4.6 (2.6–21.4)	0.81

Data are presented as mean ± standard deviation (range) or number. Abbreviations: THA, total hip arthroplasty; BMI, body mass index; OA, osteoarthritis; OFNH, osteonecrosis of the femoral head; CPO, curved periacetabular osteotomy; N/A, not applicable.

**Table 3:** Clinical evaluations.

	Osteotomy Group	Control Group	
	(*n* = 11)	(*n* = 33)	*p*
Preoperative HHS	47.5 ± 12.2 (33–66)	60.9 ± 12.6 (31–81)	0.0072 *
Postoperative HHS	94.9 ± 3.8 (86–99)	92.7 ± 12.2 (32–100)	0.99
Complications			
Dislocation	0	0	1.00
Infection	0	0	1.00
VTE	0	0	1.00
Nerve palsy	0	1 (3.0)	0.56
Revision surgery	0	1 (3.0)	0.56

Data are presented as mean ± standard deviation (range), number (%), or number. Abbreviations: HHS, Harris Hip Score; VTE, venous thromboembolism. * Significant difference: *p* < 0.05.

**Table 4:** Radiographic evaluations.

	Osteotomy Group	Control Group	
	(*n* = 11)	(*n* = 33)	*p*
McPherson’s criteria			0.40
Grade IA	10 (90.9)	32 (97.0)	
Grade IB	1 (9.1)	1 (3.0)	
Engh’s criteria			0.56
Fixation by bone ingrowth	11 (100)	32 (97.0)	
Unstable implant	0 (0)	1 (3.0)	
Cup inclination (°)	37.6 ± 8.6 (26.8–56.2)	41.4 ± 7.1 (31.9–62.5)	0.11
Cup anteversion (°)	21.3 ± 8.3 (4.5–34.8)	21.2 ± 8.3 (5.6–35.2)	0.99
Vertical distance (mm)	27.5 ± 4.6 (22.7–34.9)	24.3 ± 4.3 (16.8–36.5)	0.16
Acetabular offset (mm)	35.6 ± 2.8 (29.6–38.5)	34.0 ± 5.2 (21.3–43.5)	0.22
Femoral offset (mm)	34.5 ± 5.3 (26.2–43.9)	37.4 ± 6.6 (23.9–56.3)	0.27
Global offset (mm)	70.1 ± 7.2 (57.7–81.4)	71.4 ± 7.6 (56.4–86.0)	0.67

Data are presented as mean ± standard deviation (range) or number (%).

## Data Availability

The data that support the findings of this study are available from the corresponding author, Koichi Kinoshita, upon reasonable request.
